# A mandibular second molar with a middle mesial root canal

**DOI:** 10.1002/ccr3.2779

**Published:** 2020-03-09

**Authors:** Takayoshi Nagahara, Katsuhiro Takeda, Keinoshin Wada, Satomi Shirawachi, Tomoyuki Iwata, Hidemi Kurihara, Hideki Shiba

**Affiliations:** ^1^ Nippon Kokan Fukuyama Hospital Fukuyama Japan; ^2^ Department of Biological Endodontics Graduate School of Biomedical and Health Sciences Hiroshima University Hiroshima Japan; ^3^ Wada Dental Clinic Fukuyama Japan; ^4^ Department of Periodontal Medicine Graduate School of Biomedical and Health Sciences Hiroshima University Hiroshima Japan

**Keywords:** cone‐beam computed tomography, mandibular second molar, middle mesial canal, troughing preparation

## Abstract

The present case report describes the clinical detection and root canal management of a rare middle mesial canal of a Japanese mandibular second molar by troughing preparation using an operating microscope and cone‐beam computed tomography.

## Graphical abstract

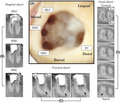

## 1 INTRODUCTION

A pulpectomy of a 44‐year‐old Japanese mandibular second molar with a rare middle mesial canal was performed using troughing preparation. In addition, cone‐beam computed tomography images revealed the morphology of the three root canals in the mesial root.

Prior knowledge of root canal anatomy is essential for the success of endodontic therapy. To remove bacteria from the root canal system and prevent reinfection, it is important to find all the main canals in the tooth root in the initial root canal treatment. Though a root canal system is, in general, complicated, an operating microscope and cone‐beam computed tomography (CBCT) are effective for analyzing root canals.^1^, ^2^

A mandibular molar commonly has two roots. In two‐rooted mandibular second molars, the most common root canal configuration is Vertucci type IV (two root canals, two apical foramina) or Type II (two root canals, one apical foramen) in a mesial root, and Type I (one root canal, one apical foramen) in a distal root.^3^ In addition, C‐shaped root canals have been found more frequently in Asian populations than in other populations.^4^, ^5^, ^6^ Internal and external configurations of teeth vary depending on, not only geographic regions, but also sex, age, ethnicity, and population group.^5^, ^7^, ^8^, ^9^, ^10^, ^11^, ^12^ All these factors need to be taken into consideration in root canal treatment.

The mesial root canals of the first and second mandibular molars do not present a consistent pattern.^13^ MM canal, whose orifice is, in general, disclosed, is sometimes located as an intermediate canal in the developmental groove connecting a mesiobuccal (MB) canal and a mesiolingual (ML) canal.^14^, ^15^ Pomeranz et al^16^ classified MM canals into three types as follows: (a) fin, allowing free instrument movement between the main and accessory canal; (b) confluent, having a separate orifice but merging more apically with the MB or ML canals; and (c) independent, having a separate orifice and apical terminus. Since Vertucci and Williams^17^ as well as Barke et al^18^ first described the presence of a MM canal in a mandibular first molar, there have been multiple subsequent reports of aberrant canal morphology in mandibular second molars.^7^ Various published articles on the presence of a MM canal in Europeans, Asians, Africans, and South/North American populations, show that the incidence of a mandibular second molar with a MM canal ranges from 0% to 12.8%.^3^, ^5^, ^7^, ^8^, ^10^, ^11^, ^12^, ^16^, ^19^, ^20^, ^21^, ^22^, ^23^, ^24^, ^25^, ^26^, ^27^, ^28^, ^29^, ^30^, ^31^, ^32^, ^33^, ^34^, ^35^, ^36^, ^37^, ^38^, ^39^, ^40^, ^41^, ^42^, ^43^, ^44^, ^45^, ^46^, ^47^, ^48^ Nosrat et al^9^ and Martins et al^5^ found significant differences in the incidence of a MM canal between Caucasian and non‐Caucasian populations. Surprisingly, our PubMed literature search did not reveal any clinical case reports or original research articles showing a MM root canal in an Asian mandibular second molar.^3^, ^5^, ^7^, ^11^, ^42^

The present case report describes the clinical detection and root canal management of a MM canal of a Japanese mandibular second molar by troughing preparation^49^, ^50^, ^51^ using an operating microscope and CBCT.

## 2 CASE REPORT

**Figure 1:**
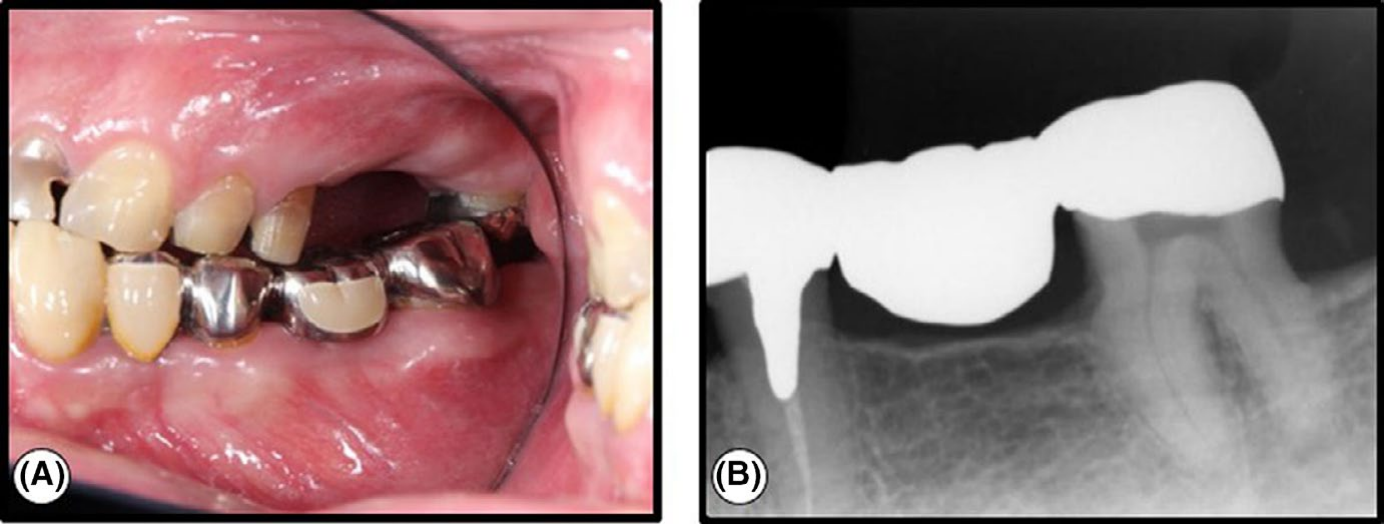
Intraoral photograph and dental radiograph of tooth 37 at the first visit. (A) Clinical view of left maxillary and mandibular. Tooth 26 and 27 were missing. Tooth 33, 35, and 37 were set on the dental bridge prosthesis. There was no vertical clearance for prosthesis, (B) Dental radiograph. A radiograph showed no specifics

A 44‐year‐old male patient with a medical history of type II diabetes and hyperlipidemia was an inpatient at Nippon Kokan Fukuyama Hospital. The patient complained of difficulty masticating and was referred to the dental department by his physician. An oral examination showed that the maxillary first and second molars were missing, and that the vertical clearance was insufficient (Figure 1A). The left mandibular first premolar, and first and third molars had been extracted due to extensive dental caries, and the 5‐unit bridge at the abutment of the left mandibular canine, second premolar and second molar was fixed approximately 10 years prior to this first visit (Figure 1A). In order to assist the patient with mastication, a left maxillary prosthesis had been planned. However, due to the insufficient vertical clearance (Figure 1A), pulpectomy at the left mandibular second molar was necessary prior to the prosthesis.

The pocket probing depth of the left mandibular second molar was 3 mm. The molar from which the full metal crown, which was a part of the 5‐unit bridge, had been removed, reacted to electric pulp vitality test using PULPER^®^ (GC) and Digitest^®^ (Parkell). A radiograph revealed no specific pathosis in the molar or the surrounding periapical and periodontal tissues (Figure 1B). Thus, the left mandibular second molar where the pulpectomy was planned, had normal pulp, dentin and periodontal tissues, including the periapical tissues. Informed consent for the pulpectomy and prosthesis was obtained.

**Figure 2:**
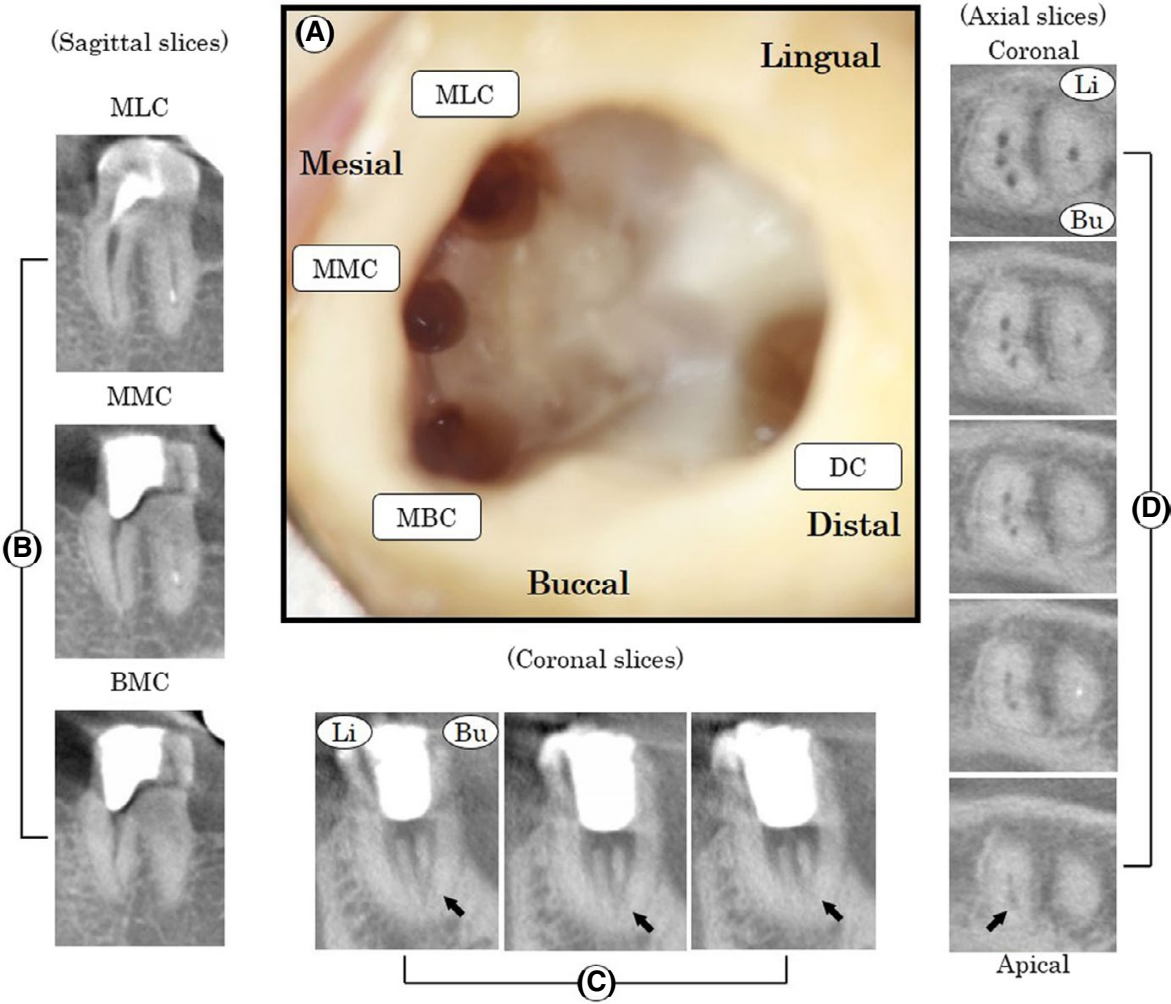
Location of root canal orifices and CBCT images after the root canal preparation. (A) Location of root canal orifices. Mesiobuccal canal; MBC, middle mesial canal; MMC, mesiolingual canal; MLC, distal canal; DC. (B, C, and D) A series of CBCT; Sagittal slices (B), coronal slice (C), and axial slices (D) clearly show MBC, MMC, and MLC. MMC joined MBC at an apical side (black arrow). Buccal; Bu, Lingual; Li

**Figure 3:**
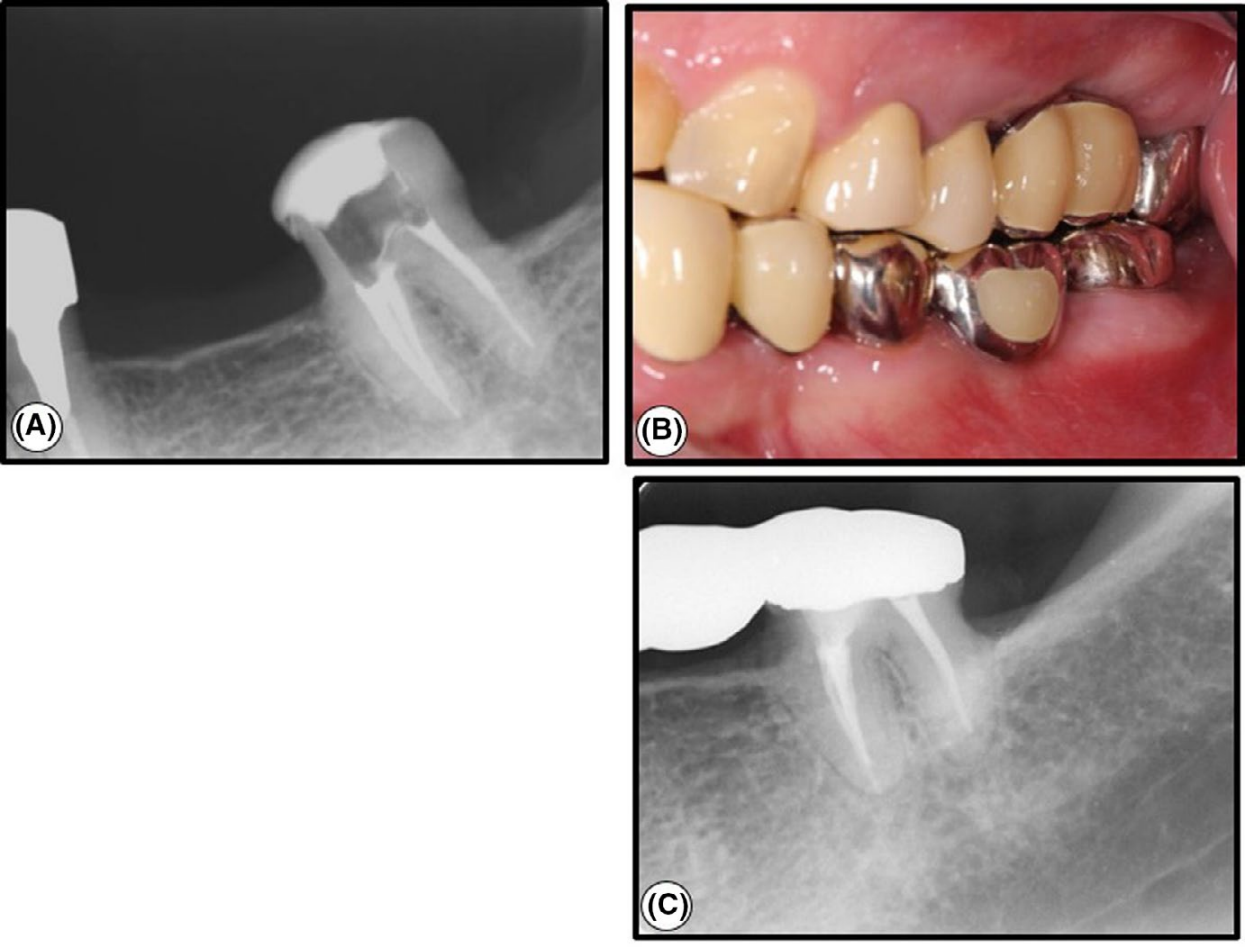
The root canal filling and one‐year follow‐up. A, Dental radiograph after the root canal filling. B, Intraoral photograph at the 1‐year follow‐up. A dental bridge was set on the tooth (maxillary bridge; the 5‐unit bridge at the abutment of tooth 24, 25, and 28. Mandibular bridge; the 5‐unit bridge at the abutment of tooth 33, 35, and 37). Clinically, the left mandibular second molar was asymptomatic and no periodontal pocket was found. C, A dental radiograph was taken at the 1‐y follow‐up. Unusual views were not seen

After disinfection with povidone iodine (Meiji Seika Pharma CO., Ltd.), Xylocaine^®^ (DENTSPLY‐Sankin Co, Ltd.) local anesthetic was administered in the left mandibular region. The left mandibular second molar was isolated with a rubber dam. After access cavity preparation, three main root canal orifices (MB canal, ML canal, and distal canal) were found under the operating microscope (ManiScope Z^®^, Mani). A protuberance from the mesial axial wall, which impaired direct access to the developmental groove between MB and ML root canal orifices, was removed using a long‐shank round bur (#2 Stainless Bur Hard^®^, Mani). After its removal, stickiness was encountered, while exploring the fissure with a sharp endodontic explorer (DG 16^®^, Hu‐Friedy). This was followed by the appearance of a bleeding point between the MB and ML canal orifices. The bleeding point, which could be negotiated with a size 8/10 K‐file (Mani), was found to be a root canal orifice of a MM canal. The four root canal orifices were shaped with Gates Glidden drills (#1 and #2, Mani). The working length was determined with a size 15 K‐file (Mani) by using the electric apex locator (Root ZX^®^; J Morita). The working length for the MB, ML, and distal canals was determined to be 16 mm. The working length for the MM canal was determined to be 15 mm. Apical preparations in the three mesial canals and the distal canal were enlarged using K‐files (Mani) to size 35 and 45, respectively. Figure 2A shows the chamber floor after the root canal enlargement. The chamber floor had three root canal orifices in the mesial and one in the distal root. The root canals were irrigated with 10% sodium hypochlorite (Neo Cleaner^®^, Neo Dental) and ethylenediaminetetraacetic acid (EDTA) (Smear Clean^®^, Nippon Shika Yakuhin KK), while calcium hydroxide (Calcipex II^®^, Nippon Shika Yakuhin KK) was employed as an intracanal medication. The access cavity was then temporarily double sealed with temporary stopping (Temporasy stopping^®^, GC Dental Industrial Corp.) and glass ionomer cement (Base cement^®^, Shofu Inc). The patient was recalled after one week, and the tooth was found to be asymptomatic. Multi‐slice CBCT scans of the left mandibular second molar were performed in order to confirm the anatomical structure of the mesial root. The four root canals were irrigated with sodium hypochlorite and EDTA to remove any remaining calcium hydroxide, and sterile paper points (Dentsplay) were placed. The cavity was then double sealed with temporary stopping and glass ionomer cement. The morphology of the second molar was obtained in coronal, axial, and sagittal sections using the CBCT images (3DX Multi‐Image Micro CT FPD8, J Morita). The CBCT images demonstrated that the left mandibular second molar had two roots (Figure 2B‐D), but not the C‐shaped root. The coronal and axial images revealed that the mesial root had three root canals (Figure 2C,D). On sagittal view, it seemed each mesial canal had been shaped to appropriate working length around each apex (Figure 2B). The three prepared canals in the mesial root in fact each originated from a separate root canal orifice, but the MB and MM canals joined apically (Figure 2C,D). This morphology of the MB, MM, and ML canals was thus termed “confluent” according to Pomeranz's classification.^16^ Since the tooth was asymptomatic, the root canals were filled with gutta‐perche (GC, GC Dental Industrial Corp.) and sealers (NISHIKA CANAL SEALER BG^®^, nishika nippon shika yakuhin Co., Ltd.) using the lateral condensation technique (Figure 3A).

The one‐year follow‐up demonstrated that the left mandibular second molar with dental bridge was asymptomatic with no pain, swelling, or tooth mobility, and a less than 3 mm probing depth (Figure 3B). The radiograph showed no pathological changes in the tooth and its surrounding tissues (Figure 3C).

## 3 DISCUSSION

Many previous in vitro and in vivo studies using CT, CBCT, micro CT, or operating microscopes have reported that the incidence of a mandibular second molar with a MM canal ranges from 0% to 12.8%.^3^, ^5^, ^7^, ^8^, ^10^, ^11^, ^12^, ^16^, ^19^, ^20^, ^21^, ^22^, ^23^, ^24^, ^25^, ^26^, ^27^, ^28^, ^29^, ^30^, ^31^, ^32^, ^33^, ^34^, ^35^, ^36^, ^37^, ^38^, ^39^, ^40^, ^41^, ^42^, ^43^, ^44^, ^45^, ^46^, ^47^, ^48^ Asian ethnic groups showed a higher prevalence of the Vertucci type I configuration, whereas Caucasian groups demonstrated Vertucci root canal configuration variability.^5^ Nosrat et al^9^ found significant differences in the incidence of a MM canal between Caucasian and non‐Caucasian groups. Our PubMed literature search did not reveal any clinical case reports or original research articles showing a MM root canal in an Asian mandibular second molar.^3^, ^5^, ^7^, ^11^, ^42^ Hence, a MM canal in the mandibular second molar is a rare phenomenon in Asian populations such as Japanese, Korean, and Chinese. To the best of our knowledge, this case report is the first to demonstrate a mesial root canal in a Japanese mandibular second molar.

MM canals have been described as having a small orifice deep within the isthmus or a developmental groove between the two orifices of the MB and ML canals.^52^ Age is associated with the presence of MM canals and small accessory canals.^8^, ^12^ Peiris et al^8^ confirmed that canal development is completed at around 30‐40 years of age in molars. Azim et al^51^ correlated the occurrence of MM canals with age and concluded that younger patients, aged 30‐40 years, had a significantly higher incidence of a MM canal. Nosrat et al^9^ reported that the incidence of MM canals was 32.1% in patients <20 years old, 23.8% in patients 21‐40 years old, and 3.8% in patients older than 40. The significantly higher incidence of a MM canal in younger patients is explained by the calcification process of the pulp, which undergoes a reduction in size due to the continuous deposition of dentin with age. Thus, a MM canal in a 44‐year‐old patient is less likely than in a younger patient.

The recent technical studies suggest that the detection and the negotiation of MM canal were increased to 22%‐77.41%^49^, ^50^, ^51^, ^52^ by using specialized instruments under the operating microscope. Their management has been minimally invasive. Especially, troughing technique may enhance the chances of locating MM canal.^51^ And then, troughing preparation is safe and effective for the negotiation of MM canals.^49^, ^50^, ^51^ In brief, troughing technique requires minimal dentin removal between the MB and ML canals in a mesio‐apical direction away from the furcal danger zone. Troughing at that level requires clear visibility, specialized instruments of small size munce discovery burs, and caution to avoid strip perforation and its subsequent using ultrasound tips or long‐shank round burs under the operating microscope. Therefore, because of the thin dentine related to the distal aspect of the MM canal and the position of the orifice, the integrity of root structure at the coronal level might be jeopardized by deep troughing if large instruments are used. Hence, further apical extension of troughing preparation might jeopardize the dentin thickness around the danger zone and possibly lead to root perforations. Several authors have suggested that a troughing preparation with a depth ranging between 0.7 and 2.0 mm is adequate.^49^, ^50^, ^51^

In conclusion, the diagnosis and management of MM canal are important, and the management should have been minimally invasive. We successfully carried out root canal treatment on an Asian mandibular second molar with a rare MM canal using troughing preparation. In addition, CBCT images revealed the “confluent” morphology of the three root canals in the mesial root.^16^

## CONFLICT OF INTEREST

None declared for all authors.

## AUTHOR CONTRIBUTION

TN, KT, KW, SS, TI, HK, and HS: drafted the manuscript and contributed to treatment of the patient. All authors have read and approved the final manuscript.

## References

[1] Hiebert BM , Abramovitch K , Rice D , Torabinejad M . Prevalence of second mesiobuccal canals in maxillary first molars detected using cone‐beam computed tomography, direct occlusal access, and coronal plane grinding. J Endod. 2017;43(10):1711‐1715. 28735796 10.1016/j.joen.2017.05.011

[2] Yoshioka T , Kobayashi C , Suda H . Detection rate of root canal orifices with a microscope. J Endod. 2002;28(6):452‐453. 12067127 10.1097/00004770-200206000-00008

[3] Kim SY , Kim BS , Kim Y . Mandibular second molar root canal morphology and variants in a Korean subpopulation. Int Endod J. 2016;49(2):136‐144. 25652228 10.1111/iej.12437

[4] Kato A , Ziegler A , Higuchi N , Nakata K , Nakamura H , Ohno N . Aetiology, incidence and morphology of the C‐shaped root canal system and its impact on clinical endodontics. Int Endod J. 2014;47(11):1012‐1033. 24483229 10.1111/iej.12256 PMC4258081

[5] Martins JNR , Gu Y , Marques D , Francisco H , Carames J . Differences on the root and root canal morphologies between Asian and white ethnic groups analyzed by cone‐beam computed tomography. J Endod. 2018;44(7):1096‐1104. 29861062 10.1016/j.joen.2018.04.001

[6] von Zuben M , Martins JNR , Berti L , et al. Worldwide prevalence of mandibular second molar C‐shaped morphologies evaluated by cone‐beam computed tomography. J Endod. 2017;43(9):1442‐1447. 28734652 10.1016/j.joen.2017.04.016

[7] Bansal R , Hegde S , Astekar M . Morphology and prevalence of middle canals in the mandibular molars: a systematic review. J Oral Maxillofac Pathol. 2018;22(2):216‐226. 30158775 10.4103/jomfp.JOMFP_194_17 PMC6097385

[8] Peiris HR , Pitakotuwage TN , Takahashi M , Sasaki K , Kanazawa E . Root canal morphology of mandibular permanent molars at different ages. Int Endod J. 2008;41(10):828‐835. 18822010 10.1111/j.1365-2591.2008.01428.x

[9] Nosrat A , Deschenes RJ , Tordik PA , Hicks ML , Fouad AF . Middle mesial canals in mandibular molars: incidence and related factors. J Endod. 2015;41(1):28‐32. 25266468 10.1016/j.joen.2014.08.004

[10] Martins JNR , Ordinola‐Zapata R , Marques D , Francisco H , Carames J . Differences in root canal system configuration in human permanent teeth within different age groups. Int Endod J. 2017;51(8):931‐941. 10.1111/iej.12896 29363147

[11] Martins JNR , Marques D , Silva E , Carames J , Versiani MA . Prevalence studies on root canal anatomy using cone‐beam computed tomographic imaging: a systematic review. J Endod. 2018;45(4):372‐386. 10.1016/j.joen.2018.12.016 30833097

[12] Martins JNR , Marques D , Mata A , Carames J . Root and root canal morphology of the permanent dentition in a Caucasian population: a cone‐beam computed tomography study. Int Endod J. 2017;50(11):1013‐1026. 27883205 10.1111/iej.12724

[13] Villas‐Bôas MH , Bernardineli N , Cavenago BC , et al. Micro‐computed tomography study of the internal anatomy of mesial root canals of mandibular molars. J Endod. 2011;37(12):1682‐1686. 22099905 10.1016/j.joen.2011.08.001

[14] Vertucci FJ . Root canal morphology and its relationship to endodontic procedures. Endod Topics. 2005;10(1):3‐29.

[15] Fabra‐Campos H . Three canals in the mesial root of mandibular first permanent molars: a clinical study. Int Endod J. 1989;22(1):39‐43. 2599659 10.1111/j.1365-2591.1989.tb00503.x

[16] Pomeranz HH , Eidelman DL , Goldberg MG . Treatment considerations of the middle mesial canal of mandibular first and second molars. J Endod. 1981;7(12):565‐568. 6985549 10.1016/S0099-2399(81)80216-6

[17] Vertucci FJ , Williams RG . Root canal anatomy of the mandibular first molar. J N J Dent Assoc. 1974;45(3):27‐28. 4523925

[18] Barker BCW , Parsons KC , Mills PR , Williams GL . Anatomy of root canals. III. Permanent mandibular molars. Aust Dent J. 1974;19(6):408‐413. 4533939 10.1111/j.1834-7819.1974.tb02372.x

[19] Ahmed HA , Abu‐bakr NH , Yahia NA , Ibrahim YE . Root and canal morphology of permanent mandibular molars in a Sudanese population. Int Endod J. 2007;40(10):766‐771. 17714468 10.1111/j.1365-2591.2007.1283.x

[20] Al‐Qudah AA , Awawdeh LA . Root and canal morphology of mandibular first and second molar teeth in a Jordanian population. Int Endod J. 2009;42(9):775‐784. 19549153 10.1111/j.1365-2591.2009.01578.x

[21] Caliskan MK , Pehlivan Y , Sepetcioglu F , Turkun M , Tuncer SS . Root canal morphology of human permanent teeth in a Turkish population. J Endod. 1995;21(4):200‐204. 7673821 10.1016/S0099-2399(06)80566-2

[22] Celikten B , Tufenkci P , Aksoy U , et al. Cone beam CT evaluation of mandibular molar root canal morphology in a Turkish Cypriot population. Clin Oral Investig. 2016;20(8):2221‐2226. 10.1007/s00784-016-1742-2 26850623

[23] de Carvalho MC , Zuolo ML . Orifice locating with a microscope. J Endod. 2000;26(9):532‐534. 11199796 10.1097/00004770-200009000-00012

[24] Demirbuga S , Sekerci AE , Dincer AN , Cayabatmaz M , Zorba YO . Use of cone‐beam computed tomography to evaluate root and canal morphology of mandibular first and second molars in Turkish individuals. Med Oral Patol Oral Cir Bucal. 2013;18(4):e737‐e744. 23524421 10.4317/medoral.18473 PMC3731107

[25] Felsypremila G , Vinothkumar TS , Kandaswamy D . Anatomic symmetry of root and root canal morphology of posterior teeth in Indian subpopulation using cone beam computed tomography: a retrospective study. Eur J Dent. 2015;9(4):500‐507. 26929687 10.4103/1305-7456.172623 PMC4745230

[26] Gulabivala K , Aung TH , Alavi A , Ng YL . Root and canal morphology of Burmese mandibular molars. Int Endod J. 2001;34(5):359‐370. 11482719 10.1046/j.1365-2591.2001.00399.x

[27] Gulabivala K , Opasanon A , Ng YL , Alavi A . Root and canal morphology of Thai mandibular molars. Int Endod J. 2002;35(1):56‐62. 11853239 10.1046/j.1365-2591.2002.00452.x

[28] Hartwell G , Bellizzi R . Clinical investigation of in vivo endodontically treated mandibular and maxillary molars. J Endod. 1982;8(12):555‐557. 6962275 10.1016/S0099-2399(82)80016-2

[29] Madani ZS , Mehraban N , Moudi E , Bijani A . Root and canal morphology of mandibular molars in a selected Iranian population using cone‐beam computed tomography. Iran Endod J. 2017;12(2):143‐148. 28512476 10.22037/iej.2017.29 PMC5431731

[30] Martinez‐Berna ABP . Investigacion clinica de molars inferiors con cinco conductos. Boletin de Informacion Dental. 1983;43:27‐41.

[31] Subha N , Minu K , Prabhakar V , Prabu M . Root canal morphology of permanet mandibular second molar in a south Indian population using computed tomography. Cons Dent Endod J. 2016;1(1):1‐5.

[32] Neelakantan P , Subbarao C , Ahuja R , Subbarao CV , Gutmann JL . Cone‐beam computed tomography study of root and canal morphology of maxillary first and second molars in an Indian population. J Endod. 2010;36(10):1622‐1627. 20850665 10.1016/j.joen.2010.07.006

[33] Neelakantan P , Subbarao C , Subbarao CV , Ravindranath M . Root and canal morphology of mandibular second molars in an Indian population. J Endod. 2010;36(8):1319‐1322. 20647088 10.1016/j.joen.2010.04.001

[34] Nur BG , Ok E , Altunsoy M , Aglarci OS , Colak M , Gungor E . Evaluation of the root and canal morphology of mandibular permanent molars in a south‐eastern Turkish population using cone‐beam computed tomography. Eur J Dent. 2014;8(2):154‐159. 24966763 10.4103/1305-7456.130584 PMC4054043

[35] Pan JYY , Parolia A , Chuah SR , Bhatia S , Mutalik S , Pau A . Root canal morphology of permanent teeth in a Malaysian subpopulation using cone‐beam computed tomography. BMC Oral Health. 2019;19(1):14. 30642318 10.1186/s12903-019-0710-z PMC6332542

[36] Pawar AM , Pawar M , Kfir A , et al. Root canal morphology and variations in mandibular second molar teeth of an Indian population: an in vivo cone‐beam computed tomography analysis. Clin Oral Investig. 2017;21(9):2801‐2809. 10.1007/s00784-017-2082-6 28281013

[37] Peiris R , Takahashi M , Sasaki K , Kanazawa E . Root and canal morphology of permanent mandibular molars in a Sri Lankan population. Odontology. 2007;95(1):16‐23. 17660977 10.1007/s10266-007-0074-8

[38] Perez‐Heredia M , Ferrer‐Luque CM , Bravo M , Castelo‐Baz P , Ruiz‐Pinon M , Baca P . Cone‐beam computed tomographic study of root anatomy and canal configuration of molars in a Spanish population. J Endod. 2017;43(9):1511‐1516. 28735786 10.1016/j.joen.2017.03.026

[39] Pineda F , Kuttler Y . Mesiodistal and buccolingual roentgenographic investigation of 7,275 root canals. Oral Surg Oral Med Oral Pathol. 1972;33(1):101‐110. 4500261 10.1016/0030-4220(72)90214-9

[40] Plotino G , Tocci L , Grande NM , et al. Symmetry of root and root canal morphology of maxillary and mandibular molars in a white population: a cone‐beam computed tomography study in vivo. J Endod. 2013;39(12):1545‐1548. 24238444 10.1016/j.joen.2013.09.012

[41] Arayasantiparb R , Wanichwetin W , Banomyoug D . Prevalence and morphology of middle mesial canals in a group of Thai permanent mandibular molars from cone‐beam computed tomography images. M Dent J. 2017;37(3):281‐287.

[42] Peiris R , Takahashi M , Sasaki K , Kanazawa E . Root and canal morphology of human permanent teeth in a Sri Lankan and Japanese population. Odontology. 2007;95:16‐23. 17660977 10.1007/s10266-007-0074-8

[43] Sert S , Aslanalp V , Tanalp J . Investigation of the root canal configurations of mandibular permanent teeth in the Turkish population. Int Endod J. 2004;37(7):494‐499. 15189440 10.1111/j.1365-2591.2004.00837.x

[44] Sert S , Bayirli GS . Evaluation of the root canal configurations of the mandibular and maxillary permanent teeth by gender in the Turkish population. J Endod. 2004;30(6):391‐398. 15167464 10.1097/00004770-200406000-00004

[45] Skidmore AE , Bjorndal AM . Root canal morphology of the human mandibular first molar. Oral Surg Oral Med Oral Pathol. 1971;32(5):778‐784. 5286234 10.1016/0030-4220(71)90304-5

[46] Tahmasbi M , Jalali P , Nair MK , Barghan S , Nair UP . Prevalence of middle mesial canals and isthmi in the mesial root of mandibular molars: an in vivo cone‐beam computed tomographic study. J Endod. 2017;43(7):1080‐1083. 28527840 10.1016/j.joen.2017.02.008

[47] Torres A , Jacobs R , Lambrechts P , et al. Characterization of mandibular molar root and canal morphology using cone beam computed tomography and its variability in Belgian and Chilean population samples. Imaging Sci Dent. 2015;45(2):95‐101. 26125004 10.5624/isd.2015.45.2.95 PMC4483626

[48] Vertucci FJ . Root canal anatomy of the human permanent teeth. Oral Surg Oral Med Oral Pathol. 1984;58(5):589‐599. 6595621 10.1016/0030-4220(84)90085-9

[49] Keles A , Keskin C . Detectability of middle mesial root canal orifices by troughing technique in mandibular molars: a micro‐computed tomographic study. J Endod. 2017;43(8):1329‐1331. 28606668 10.1016/j.joen.2017.03.021

[50] Karapinar‐Kazandag M , Basrani BR , Friedman S . The operating microscope enhances detection and negotiation of accessory mesial canals in mandibular molars. J Endod. 2010;36(8):1289‐1294. 20647082 10.1016/j.joen.2010.04.005

[51] Azim AA , Deutsch AS , Solomon CS . Prevalence of middle mesial canals in mandibular molars after guided troughing under high magnification: an in vivo investigation. J Endod. 2015;41(2):164‐168. 25442720 10.1016/j.joen.2014.09.013

[52] Chavda SM , Garg SA . Advanced methods for identification of middle mesial canal in mandibular molars: an in vitro study. Endodontology. 2016;28(2):92‐96.

